# Supplementary material for: West Nile Virus and Usutu Virus: A Post-Mortem Monitoring Study in Wild Birds from Rescue Centers, Central Italy
Source: Viruses. 2022 Sep 9;14(9):1994. doi: 10.3390/v14091994 (PMC9503110; doi:10.3390/v14091994)
Supplement: Supplementary file 1 [file viruses-14-01994-s001.zip › viruses-1876938-supplementary.pdf]

Birds were examined in various percentages in the different seasons of the year, such as: Winter (December, January, February), Spring (March, April, May), Summer (June, July, August), Autumn (September, October, December). Detailed data regarding birds collected in each season for each year are reported in Table S1. Additionally, a graphical representation of the data is reported in Figure S1.

Table S1. Summary of the birds examined for each season in each year of monitoring.

	Autumn	Winter	Spring	Summer	Not available (month)
**2017**	10	13	0	0	1
**2018**	46	44	3	149	2
**2019**	16	42	49	54	40
**2020**	0	3	5	76	0
**2021**	0	0	2	0	0
**Not available (year)**	0	0	0	0	21
**Total**	72	102	59	279	64

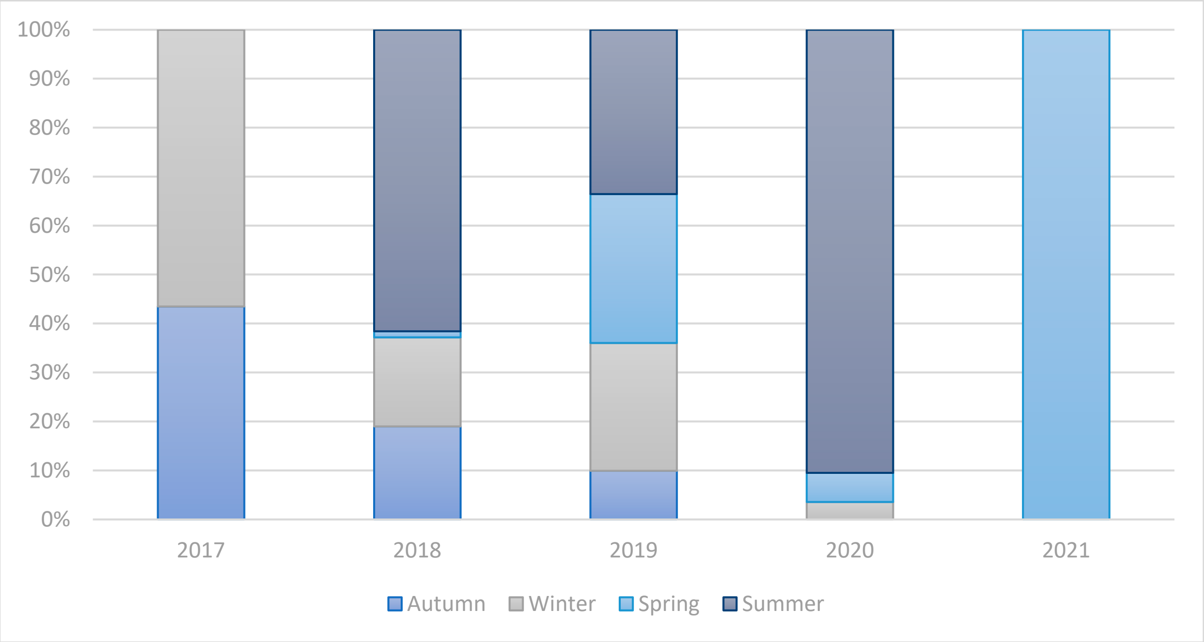

Figure S1. Clustered bar chart on the percentages of birds examined in each season per year. For the year 2017 and 2021 the distribution of the birds examined for each season was affected from the starting and ending period of the study. For the year 2019, year of detection of WNV in the little grebe, the monitoring was more uniformly distributed throughout the four seasons. In general, for 2018, 2019, and 2020, most birds were examined in autumn and summer.

Of the total of birds examined (n = 576), the majority was examined in Umbria (n = 405), followed by Tuscany (n = 155) and Latium (n = 16), respectively. A graphical representation of those data is reported in Figure S2.

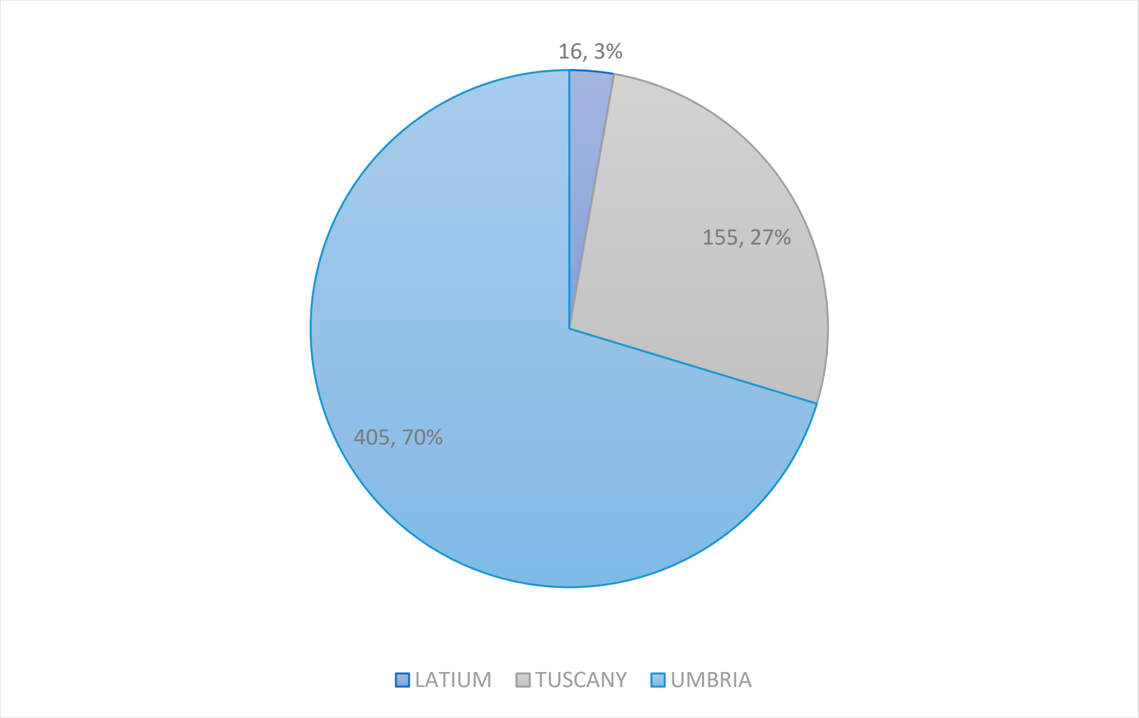

Figure S2. Pie chart on the number and percentages of birds examined in each region. The majority of birds examined were collected in Umbria, site of the were the post-mortem investigation and sampling was performed.
